# Benefits and challenges of telemedicine for heart failure consultations: a qualitative study

**DOI:** 10.1186/s12913-023-09872-z

**Published:** 2023-08-10

**Authors:** Arvind Singhal, Jillian P. Riley, Martin R. Cowie

**Affiliations:** 1 Royal Brompton Hospital, London, UK; 2 Imperial College, London, UK; 3 King’s College London, London, UK

**Keywords:** Heart failure, Telemedicine, Remote consultation, Digital health, Qualitative

## Abstract

**Background:**

Prior to the Covid-19 pandemic, heart failure (HF) disease management programmes were predominantly delivered in-person, with telemedicine being uncommon. Covid-19 resulted in a rapid shift to “remote-by-default” clinic appointments in many organisations. We evaluated clinician and patient experiences of teleconsultations for HF.

**Methods:**

From 16th March 2020, all HF appointments at a specialist centre in the UK were telemedicine-by-default through a mixture of telephone and video consultations, with rare in-person appointments. HF clinicians and patients with HF were invited to participate in semi-structured interviews about their experiences. A purposive sampling technique was used. Interviews were conducted using Microsoft Teams®, recorded and transcribed verbatim. Narrative data were explored by thematic analysis. Clinicians and patients were interviewed until themes saturated.

**Results:**

Eight clinicians and eight patients with HF were interviewed before themes saturated. Five overarching themes emerged: 1) Time utilisation – telemedicine consultations saved patients time travelling to and waiting for appointments. Clinicians perceived them to be more efficient, but more administrative time was involved. 2) Clinical assessment – without physical examination, clinicians relied more on history, observations and test results; video calls were perceived as superior to telephone calls for remote assessment. Patients confident in self-monitoring tended to be more comfortable with telemedicine. 3) Communication and rapport – clinicians experienced difficulty establishing rapport with new patients by telephone, though video was better. Patients generally did not perceive that remote consultation affected their rapport with clinicians. 4) Technology – connection issues occasionally disrupted video consultations, but overall patients and clinicians found the technology easy to use. 5) Choice and flexibility – both patients and clinicians believed that the choice of modality should be situation-dependent.

**Conclusions:**

Telemedicine HF consultations were more convenient for patients, saved them time, and were generally acceptable to clinicians, but changed workflows, consultation dynamics, and how clinical assessment was performed. Telemedicine should be used alongside in-person appointments in a “hybrid” model tailored to individual patients and settings.

## Introduction

Heart failure (HF) management programmes, including regular clinical review, are recommended by European guidelines to reduce morbidity and mortality [1]. In 2020, community transmission of Covid-19 prompted healthcare systems across the world to re-organise outpatient care in order to comply with national government restrictions, minimise the risk of transmission to patients, and to allow redeployment of staff [2]. In the United Kingdom (UK), people aged over 70 or with specific health conditions, including HF, were classed as “clinically vulnerable” owing to an increased risk of severe illness from Covid-19, and were advised to avoid all non-essential contact with non-household members, including for routine medical care [3]. For many organisations, this necessitated a dramatic shift from in-person to remote delivery of specialist outpatient care in order to continue providing disease management programmes [4, 5]. The 2021 update to the European Society of Cardiology guideline for HF highlighted some of the potential benefits of remote patient care, including reduced patient travel costs and being more in keeping with a “green” agenda, but also pointed out that there remain major gaps in evidence [1].

Prior to the Covid-19 pandemic restrictions, the use of telemedicine consultations was rare in secondary care; a report by the Organisation for Economic Co-operation and Development (OECD) found that telemedicine consultations were less than 0.2% of in-person consultations in surveyed countries, including Australia, Canada and Portugal [6]. Consequently, there are few data on the experience of HF patients (and their healthcare professional advisers) undergoing telemedicine consultation instead of in-person appointments.

As Covid-19 becomes endemic, healthcare services must plan for “the new normal” where universal face-to-face clinic reviews are likely replaced by a “blended” model of care. A qualitative evaluation of telemedicine consultations, from both the patient and healthcare professional perspective is likely to inform discussion around the optimal delivery of future outpatient care.

The Royal Brompton Hospital provides specialist HF care to patients from across the South of England. Prior to Covid-19, most patients with HF were seen 6-monthly with face-to-face appointments, weights, and blood pressure measurements, in line with national guidance [7]. It shifted to a “remote-by-default” model for all HF clinic appointments from 16^th^ March 2020, just prior to the first national “lockdown”, with face-to-face appointments only in exceptional circumstances. We aimed to evaluate the experiences of clinicians and patients who had participated in telemedicine consultations, contrasting that with their experience of the traditional “face-to-face” model of care.

Methods.

The Royal Brompton Hospital runs a specialist HF service led by five consultants and three clinical nurse specialists. All scheduled HF clinic appointments for new patients and for routine follow-up were initially converted to telephone appointments, and from July 2020 onwards the option of video consultation was made available. Video consultations were conducted using the AttendAnywhere® web-based video consultation platform – an NHS recommended platform not requiring additional software to be installed by either the patient or healthcare professional. The platform uses a virtual “waiting area” where clinicians can see which patients have logged on and are waiting, with clinicians then triggering patient access into the virtual clinic room.

Study design.

We used a qualitative study design, following the Standards for Reporting Qualitative Research [8]. Clinicians and patients were invited to participate in semi-structured interviews about their experiences of telemedicine clinics. Semi-structured interviews are defined as being “organized around a set of predetermined open-ended questions, with other questions emerging from the dialogue between interviewer and interviewee/s.” [9].

Five prompt questions guided the interview. Questions were chosen to be open-ended but ensuring key areas of interest were covered:

1. Please describe your experience of telemedicine consultations.
2. How did you find the technology?
3. What was your experience of the clinical interaction?
4. How could the experience of consultations be improved?
5. How would you like future consultations to be conducted, and why?

Data collection.

A purposive sampling technique was used for clinician and patient recruitment. Members of the HF clinical team (except for the investigators) were invited to take part in the study by e-mail and selected to ensure a good mixture of doctor and nurse responses with a range of levels of clinical experience and familiarity with technology. By the point at which interviews saturated, however, all permanent members of staff (excluding the investigators) had been interviewed, and so limited demographic data are provided to avoid identification of responses. Patients who had a scheduled appointment in either a consultant or nurse-led HF clinic between 1st January 2021 and 28th April 2021 were sent a text message link to a survey in which they were invited to participate in interviews. Patients were screened to confirm a diagnosis of HF and recent clinic attendance. Purposive sampling criteria for patients were chosen to ensure patients were representative of the wider cohort in age, sex and ethnicity, whilst also including a range of respondents. In the Royal Brompton Hospital, the median age of patients under follow-up with HF is 68 (with most aged between 45 and 85), a majority of patients are male, and the largest ethnic group is white-British. Patients were also sampled to explore emerging hypotheses; we included patients with both recent and long-term diagnoses, and a mixture of those who had video and telephone follow-up. Participants were selected and interviewed sequentially until data were “saturated” (see below).

All interviews were conducted by AS following training by JPR. Interviews were conducted and recorded using Microsoft Teams®, and then transcribed verbatim. Interviews with clinicians were conducted between 24^th^ February 2021 and 17^th^ March 2021, and interviews with patients were conducted between 9^th^ April 2021 and 19^th^ May 2021. All interviews lasted no more than 40 min.

### Data analysis

Interview transcripts were pseudo-anonymised and narrative data were analysed by thematic analysis [10, 11]. Briefly, this is an inductive process whereby data are described and organised using codes, and transcripts are compared iteratively, identifying patterns in the data through which themes emerge. The first two interview transcripts for both clinicians and patients were co-analysed by AS and JPR to ensure consistency and rigour, and emerging themes were identified and agreed on. Later interviews explored emerging themes in greater detail whilst still being receptive to new themes. Study recruitment was planned to end once themes reached “saturation”, i.e., when themes were fully developed and additional interviews did not lead to new themes [12, 13]. Microsoft Word was used for storage and analysis of transcripts.

Ethics.

The study was registered as the “VIDEO-HF” study with the UK Integrated Research Application System (IRAS number 284625) and received ethical approval from South West—Frenchay Research Ethics Committee (20/SW/0096).

## Results

Between 16^th^ March 2020 and 15^th^ March 2021 there were 2797 HF clinic appointments, of which 2761 (98.7%) were by telemedicine. Most telemedicine consultations were by telephone rather than video call, but it was not possible to gather an accurate breakdown as consultations were not coded separately in hospital IT systems.

Four consultant cardiologists in the HF service, three specialist nurses that were part of the multi-professional team, and one rotational training-grade doctor were interviewed. Half of interviewees were male and half were female. Clinician recruitment was terminated following these eight interviews as themes were deemed to have saturated.

1296 patients were sent a text message link to the survey, and 128 patients (9.9%) completed the survey. 63 respondents (49%) reported that their most recent clinical interaction was by telephone, 40 (31%) by video call, and 25 (20%) face-to-face. Using purposive sampling, we selected patients based on their clinical and social characteristics described in the methods (Table 1). Only one patient declined interview after being approached. Patients’ follow-up duration for HF ranged from 4 months to 10 years. Themes were deemed to be saturated following eight interviews.

**Table 1:** Details of patients interviewed for study

Patient	Age at interview	Sex	Patient-reported modalities used
P01	57	Male	Video and telephone
P02	65	Male	Video and telephone
P03	70	Male	Telephone
P04	56	Female	Telephone
P05	63	Female	Video and telephone
P06	75	Male	Telephone
P07	45	Female	Telephone
P08	72	Male	Video and telephone

Interviews lasted between 25 and 40 min, and transcripts were typically around 5000 words.

Five overarching themes emerged from interviews:

- Time utilisation
- Clinical assessment
- Communication and rapport
- Technology
- Choice and flexibility

Theme 1: Time utilisation.

Clinicians described how telemedicine changed how they spent their time in a clinic. There was a mix of opinions as to whether telemedicine resulted in net time cost or benefit for clinicians. They perceived that consultations were overall shorter, and time was saved by not performing physical examinations, and less time was spent between consultations waiting for patients. Some clinicians perceived telemedicine consultations to be more “efficient”:

*“Overall, I think they are shorter in my opinion…. I find that that's probably because I'm more efficient on the phone…. you get straight to the point on the telephone sometimes, or on video with some patients.”*

– S08, HF Consultant

Telemedicine also allowed the possibility of clinicians working from home and saving a journey into hospital, which could save time and be more convenient for staff.

However, clinicians perceived that more administrative time was required for appointments. One HF nurse perceived a significant increase in workload, in large part due to the time taken to obtain external results from the General Practice (GP, where patients now predominantly had blood tests) owing to the lack of interoperability between GP and hospital health records. Others perceived that more preparation was required for clinics as patients needed to be pre-allocated to clinicians to call them.

Most patients perceived that a key advantage of telemedicine consultations was that they saved them time, effort and expense of travelling to hospital:

*“That's the big benefit for me, I think, just the saving in time and expense of travel. It takes me 2, 2 1/2 hours to get there from home, so it saves me a good half day, plus the train fare. So yeah, I can see very real benefits.”*– P03, 70M

For patients who previously drove to their appointments, there was also the added benefit of not having to “burn up a load of fossil fuels, [and] get frustrated in the traffic…. it’s saving the environment”. Most patients preferred waiting at home for the video consultation rather than waiting in a hospital waiting room, as they found it more comfortable, and were able to do other things whilst they waited.

Patients did not perceive that telemedicine consultations were shorter, brief or rushed, but some patients thought that it would be more efficient for clinicians.

Theme 2: Clinical assessment.

Telemedicine consultations meant that different methods of information gathering were required to assess patients and make clinical decisions.

The lack of physical examination was deemed by most clinicians to be a significant downside of telemedicine consultations:

*“The examination can often pick up something and I don't find it very satisfactory to have someone sort of use the camera to go ‘here are my legs look at my oedema’ or not.”*

– S05, HF Consultant

Examination of oedema was possible by video but perceived to be less reliable than a physical examination:

*“At least you can, you know, turn the camera around so you can have a look and see what- what that person's legs look like, so it's a bit of a halfway house in that sense in terms of your clinical assessment.”*– S03, Training grade doctor

Clinicians stated that patient self-monitoring data, such as weight, blood pressure and heart rate measurements assisted clinical assessment. Some patients were able to also get blood tests from the community. Without physical examination and when patients did not have easy access to monitoring equipment, clinicians became more reliant on the patient history.

Patients also found that the clinical assessment in telemedicine consultations differed from in-person consultations. Physical examination was not possible by telemedicine, but most patients did not perceive this to be a problem for them.

Most patients thought that physical examination was not a vital part of the heart failure consultation, particularly if they were symptomatically stable:

*“Where it is a straightforward review of the medication, how am I, checking on blood pressure, checking those sorts of things that can be done at arm’s length, then [video consultation] is perfect.”*– P08, 72M

One patient did find routine physical examination and regular in-person clinical assessment more reassuring, and this was her primary reason for preferring face-to-face consultations.

Patients stated that telephone consultations were particularly reliant on self-described clinical status, and therefore open to a possible “mismatch” between the patient’s and clinician’s perceptions of their clinical status, whereas video allowed a visual assessment which may be able to detect this mismatch:

*“Perhaps there might be a mismatch between them saying ‘I'm absolutely fine, absolutely fine’ and you may think you don't look perhaps, you know- don't look as well, perhaps, as you're saying.”*– P01, 57M

Most patients were confident that they were able to notice any deterioration in their cardiac status and could therefore alert the clinician. Those that described being less confident in their ability to self-assess preferred in-person reviews by clinicians for routine heart failure monitoring. For example, a 56-year-old diagnosed with heart failure two years before the pandemic stated:

*“I think that sometimes that you- you could miss something yourself that- that they wouldn’t.”*– P04, 56F

Some patients found getting blood tests at GP surgeries more convenient than in hospital as it was a quicker process with shorter travel times.

Theme 3: Communication and rapport.

Clinicians stated that there was a difference in “chemistry” between in-person and remote consultations that altered the clinician-patient relationship:

*“There is a chemistry you have when there's another person next to you, another person in any room … the intimacy and the confidence of being in the close presence of some- someone else is all part of how they come to trust you and listen to you.”*– S01, HF Consultant

Clinicians almost unanimously agreed that new patients were better seen face-to-face to develop a “connection” which was felt to be important for patients to trust clinicians and thus speak freely, but also for clinicians to best interpret the significance of patient histories. The personal aspect of face-to-face consultation was thought to be particularly important when having a difficult conversation around a sensitive topic; breaking bad news or advanced care planning was thought to be inappropriate by telemedicine.

Like clinicians, patients found that telemedicine consultations changed how they communicated, affecting rapport and consultation dynamics. Patients also emphasised the importance of non-verbal communication:

*“I think just being face-to-face and closer to someone you pick up more on certain nuances with communications, and body language, that sort of thing. And it's all part of communication, isn't it? And, being in a room, you see all that.”*

– P02, 65M

Many patients described communication in video consultations to be similar to in-person consultations, as it was still possible to make use of visual cues through a screen:

*“It's pretty much the same, again it's all to do with seeing someone. I think the operative word there is seeing.”*– P02, 65M

For telephone, some patients perceived that the lack of these key visual cues impaired communication and detracted from the experience of telephone consultations. Others, however, judged that consultation modality had minimal impact on rapport.

Unlike clinicians, however, patients did not express a specific preference for first appointments to be face to face, and most respondents thought that meeting someone for the first time by telemedicine was similar to in-person appointments. Most patients stated that sensitive topics such as breaking bad news were best discussed in-person so that patients could have adequate support:

*“I think if it was like you've got six months to live, I’d probably rather do that face-to-face than over a Zoom call. I think that would be quite hard to hear that when you're on your own in the house, you know, and there's no support.”*

– P05, 63F

Theme 4: Technology.

Occasionally, technical disruptions would occur on the video platform, often due to internet connection issues, which could have a significant impact on the timing of the clinic. Clinicians described how they would then revert to telephone. Most clinicians had experienced some technical difficulties during a telemedicine consultation:

*“And when it crashes, oh my goodness, that's a disaster. You know, when you can't use it, that's quite stressful.”* – S06, HF Nurse

Video consultations required access to a computer with a webcam, personal headset and high-speed internet, ideally in a private space; such spaces were sometimes difficult to find, and computers were not always set up properly.

A disadvantage of video consultation was that occasionally latency issues (i.e., the delay between transmission of information and receipt on the other end) resulted in disrupted and staggered conversational flow:

*“When you speak, it's clear that there's a one, you know, fraction to one second delay, and there’s the risk of speaking over each other.”*– S05, HF Consultant

Some clinicians believed that telemedicine posed privacy challenges; patients and clinicians may not want others to see their home environment, and unlike in a consultation room, it was not always clear who was able to see or hear the consultation.

Most patients interviewed had prior experience of video calling outside of healthcare, often using platforms such as Zoom™ to connect with family and friends, particularly during the pandemic, and some patients had also used video platforms for work. Patients did not report technical disruptions during their video consultations, though one patient reported signal disruption in a telephone consultation.

Some patients were apprehensive before their first appointment using video technology as they were unfamiliar with the platform and concerned about potential difficulties in accessing it. Despite this, most patients found video consultations intuitive and easy-to-use:

*“It was absolutely fine. Worked- worked first time, no delays getting in and I didn't have to download anything. So that was- it was part- perfectly fine, yeah.”*

– P01, 57M

Theme 5: Choice and flexibility.

Clinicians deemed that the choice of modality was very much dependent on the patient and on the situation. Patient choice was essential, as some patients may not be comfortable with using telemedicine or have the necessary equipment for video consultations:

*“I think it's really important to give patients the choice, 'cause we are tech savvy and happy with either, but they, I think, fall into different groups…”*– S05, HF Consultant

Clinicians also had preferences based on the purpose of the consultation and needs of the patient. Where patients described worsening symptoms, the clinician stated a preference for in-person appointments to make a full assessment including physical examination. On the other hand, more “routine” appointments such as periodic assessment and conveying test results could be done by telemedicine.

Clinicians perceived that patients and healthcare systems were more receptive to the idea of telemedicine consultations owing to the Covid-19 pandemic. Clinicians deemed that clinics were unlikely to return to how they were before Covid-19, and that a mix of in-person and telemedicine consultations would likely be the future model of care:

*“So, I think the good news is it's here to stay. How do we deliver it and make sure that … all the stakeholders … get the best of this, I think we're still learning, aren't we?”*– S05, HF Consultant

Some clinicians, however, expressed fears of top-down diktats for future delivery of care, with rigid policies such as quotas for telemedicine patients, restricting flexibility for how clinicians choose to run their own clinic, or not allowing patients to choose their mode of appointment:

*“The health service has got a great tradition of making up its mind as to what the patient thinks, and then giving it back to them…”*– S01, HF Consultant

Similarly, patients stated that the choice of consultation modality depended on the situation. Most patients thought that a combination of in-person and telemedicine clinics would be ideal for future care. Communicating test results, for example, was generally perceived to be best done by telemedicine, whereas, as reported above, patients tended to prefer in-person appointments when their symptoms deteriorated, and would also prefer in-person appointments if they were receiving bad news.

Some patients thought that telemedicine should be the default modality for them, and in-person appointments should be reserved for when physical assessment was required:

*“Well, if there's no need for, uh, contact, physical contact, then obviously you don't really need to travel.” *– P02, 65M

Some patients believed that in-person appointments should be reserved for when specialist investigation was required, but otherwise telemedicine was preferable. Others, however, preferred in-person assessment as the default as the reassurance of routine physical assessment outweighed the benefits of saving a journey:

*“Well, I'd rath- I would rather come in and see the consultant myself.… I- personally I don’t mind travelling to go there.” *– P04, 56F

One patient, however, believed that the efficiency of telemedicine needed to be balanced against access to services, and he emphasised the importance of improving access:

*“Obviously, there are issues about sort of connectivity and people who don't have access to digital communication… I think it's that balance of if it's more efficient for everybody, uh, then… encouraging people towards the most efficient way is- is better.”*

– P01, 57M

## Discussion

We identified five main themes in patients’ and clinicians’ perceptions of telemedicine consultations for HF: time utilisation, clinical assessment, communication and rapport, technology, and choice and flexibility.

Comparison of clinician and patient perceptions.

Whereas clinicians had mixed opinions on whether telemedicine saved time, patients were unequivocal that telemedicine consultations saved a significant amount of journey time and were more convenient. Both patients and clinicians emphasised the importance of non-verbal communication and visual cues for clinical assessment, however clinicians placed more emphasis on the effect this had on rapport; some patients even reported that telemedicine consultation did not change rapport at all. Clinicians unanimously thought that it was best to meet patients for the first time in-person rather than by telemedicine, but patients interviewed did not express a preference for this. Patients and clinicians agreed that breaking bad news was best done in person. Most clinicians raised the issue of lack of physical examination being a limitation to telemedicine, whereas patients had mixed opinions; some patients did not perceive physical examination to be an important part of clinical assessment. Most clinicians interviewed experienced technical difficulties during video consultations and some perceived that patients would find video consultations technically challenging. Patients, on the other hand, tended to have good experiences with video consultation and did not encounter technical issues; this may simply reflect the fact that clinicians had performed a far higher number of consultations. Both patients and clinicians stated that telemedicine consultations may be more efficient.

Benefits and challenges of telemedicine.

Patients and clinicians in our study identified several benefits and challenges (summarised in Table 2) to telemedicine HF consultations compared with traditional in-person appointments.

**Table 2:** Benefits and challenges relating to telemedicine consultations as identified by interviewed clinicians and patients

	**Benefits**	**Challenges**
Face-to-face	• Better rapport • Physical examination • Easy access to same-day investigations	• Long travel times (and environmental impact of travelling) • Long waiting times • Longer consultations • Dedicated physical consultation space required
Telemedicine	• No travel necessary • Flexibility • Shorter consultations • Shorter wait for patients • Some visual assessment possible by video • No dedicated physical clinic rooms required	• More administrative time • Less rapport (particularly telephone) • Limited examination – more reliant on history • No access to same-day investigations • Need for personal computers for clinicians and high-quality internet • Need for private spaces

Comparison with prior research.

Patients in our study reported significant time benefits of telemedicine consultation. This is consistent with interviews of primary care patients in the USA [14], New Zealand [15], and the UK [16], where patients also reported benefits of reduced travel time and noted decreased wait times compared with face-to-face appointments. Clinicians in the present study perceived telemedicine consultations were overall time neutral, in keeping with findings from Donaghy et al*.* in primary where they also used the same NHS video platform (Attend Anywhere™) [16]. In their study, patients and clinicians also spoke of the advantages of picking up non-verbal cues by video which reduced miscommunication and improved rapport compared with audio-only telephone. Those interviewed also reported that serious issues or delivering bad news would be more appropriate for face-to-face consultations, consistent with the present study. Clinicians’ perceptions of video examination are consistent with findings of a recent qualitative study of primary care consultations in Sweden [17], and strategies used to examine patients for oedema by video (including using home monitoring equipment) are similar to those described previously in HF patients [18], Challenges in telemedicine consultations identified by our study participants are similar to those identified by Greenhalgh et al*.* in a hermeneutic review of the literature of HF and telehealth [19],

The VOCAL study evaluated telemedicine consultations for diabetes and cancer surgery at an acute hospital in the UK using mixed methods [20], Implementing telemedicine consultations was found to be more complex and challenging than anticipated; in contrast, our organisation and clinicians were able to transition rapidly to telemedicine, likely owing to the absolute necessity resulting from Covid-19 related social restrictions.

Discussion of methodology and limitations.

Study design.

Semi-structured interviews are the most common form of interview used in qualitative healthcare research [21]. Nonetheless, there are some limitations to this approach.

Interviewee perceptions of the interviewer, and the relationship of the interviewer to the study group may influence responses [22]. Patient interviewees were screened to ensure they had no prior medical interaction with the interviewer, but clinicians were familiar with the interviewer. Interviews are also subject to “social desirability bias” whereby respondents may be more inclined to express views they think are more acceptable to the interviewer [23].

Thematic analysis, as with all qualitative research, is subjective and subject to different interpretations of data. Two independent investigators agreed the emerging themes accurately reflected narrative data, one of whom (JPR) is an experienced researcher in qualitative methodology.

A total of 16 participants (8 clinicians and 8 patients) were interviewed before themes saturated. Although the sample size may appear small, meta-research of qualitative studies suggests this is in-fact a reasonable sample size, and that the majority of key themes emerge within the first 6 interviews [24–26].

Study population.

The Royal Brompton Hospital is a tertiary centre reviewing patients from a wide geographical area across Southern England, and patients’ and clinicians’ experiences may not be representative of those in district general hospitals, or from other countries.

Patients were recruited by a text-message link to a survey. The response rate was just under 10%, and it is likely that respondents may have been more motivated or interested in research and more positive about technology than those who did not agree to take part in the study. A strength of our study, however, is that unlike other telemedicine research pre-Covid-19 [18, 27], nearly all HF patients in the wider cohort had appointments by telemedicine, thus participants who were interviewed had not been pre-selected on the basis of their digital enthusiasm. The average age of patients interviewed in this study was 63, with the average age of HF patients at the Royal Brompton Hospital being 68, significantly younger than the national average age at diagnosis of HF of 77 in the UK [28], Familiarity with, and access to, technology generally declines with increasing age [29], and this cohort may therefore have been more digitally literate than the wider HF population. Finally, we were unable to assess the views of primary care clinicians, who are likely to have been impacted by the shift towards telemedicine, which may have resulted on increased demands on local investigations such as blood tests.

Significance and implications.

Whilst the rapid transition to telemedicine consultations was “catalysed” by Covid-19 related social restrictions and “lockdowns”, healthcare systems have recommended expanding telemedicine in order to help service over-burdened hospital outpatient departments [5, 30]. The 2021 updated ESC guideline for HF recommended home telemonitoring as an option for follow-up of chronic HF patients; importantly they state that this need not be superior to usual care for this approach to be adopted [1]. Our findings show that telemedicine is acceptable for many routine HF care decisions when in-person appointments are not possible, and may even be favoured compared with face-to-face clinics in certain circumstances such as routine follow-up or relaying test results. A flexible, “hybrid” approach, tailoring the delivery of care to the needs of the patient, could provide the best of both worlds by using the right tool at the right time. Delivering care by telemedicine for stable HF patients who are comfortable using technology frees up in-person clinic capacity for patients in greater need of a face-to-face assessment. In order to sustain the use of telemedicine, it is vital to address the challenges raised by telemedicine users. To save clinicians time requesting local test results from GPs, better integrated IT systems are needed, allowing easier sharing of health data across providers. Easier access to self-monitoring equipment, such as home blood pressure, oxygen saturation and heart rate monitors could lessen the impact of the lack of physical examination. Higher quality internet connections and software improvements may improve latency and virtual consultation quality; hospitals may consider investing in dedicated telemedicine equipment.

Further research should focus on safety and outcomes, and cost–benefit analyses of telemedicine consultation for HF care for telemedicine strategies. Other areas of potential research include whether telemedicine may allow more flexible working to help address staff retention and shortages. Additionally, clinicians reported increased reliance on local blood tests and imaging. It is therefore important to assess the impact of this on primary care resources and demand for specialist imaging.

## Conclusion

Telemedicine consultations change clinician workflows and consultation dynamics but are likely to play an important ongoing role in outpatient HF care as they are more convenient for patients and save them time; they are particularly suitable for patients who are clinically stable. For patients reporting a deterioration in symptoms or for those less confident in self-monitoring, clinical assessment is more challenging via telemedicine and so the option for in-person assessment remains necessary. Choice and flexibility are key to a positive experience, and patients and staff should be supported to ensure they are able to get the best experience from their consultation [31]. A hybrid model allowing both types of appointments allow organisations to tailor HF care to patients’ needs and resources.

## Data Availability

Aggregated data are available from the corresponding author on reasonable request. Full datasets (i.e., interview transcripts) are not publicly available to minimise the possible identification of participants.

## Abbreviations

GP: General Practice/Practitioner
HF: Heart Failure
IT: Information Technology
NHS: National Health Service
OECD: Organisation for Economic Co-operation and Development
UK: United Kingdom
USA: United States of America
